# The prevalence of 30 HPV genotypes detected by EUROArray HPV in cervical samples among unvaccinated women from Vojvodina province, Serbia

**DOI:** 10.1371/journal.pone.0249134

**Published:** 2021-04-14

**Authors:** Gordana Kovacevic, Vesna Milosevic, Natasa Nikolic, Aleksandra Patic, Nela Dopudj, Jelena Radovanov, Ivana Hrnjakovic Cvjetkovic, Vladimir Petrovic, Milena Petrovic

**Affiliations:** 1 Institute for Public Health Vojvodina, Novi Sad, Serbia; 2 Faculty of Medicine, University of Novi Sad, Novi Sad, Serbia; 3 Merck Sharp & Dohme D.O.O., Belgrade, Serbia; University of Nebraska-Lincoln, UNITED STATES

## Abstract

This study evaluates the pre-vaccination prevalence of HPV infection in women from Vojvodina, Serbia, according to age and cytological status. A total of 1,495 women, ranging from 18 to 65 years of age, with different cytological results were enrolled. The HPV genotyping assay was performed using the EUROArray HPV test in order to detect thirty genitally relevant HPV subtypes. In our study, the most prevalent genotypeswere HPV 16, 31, 51, and 53. Among these, HPV 16 was consistently present in all cytological subgroups. Twelve HPV genotypes classified as carcinogenic to humans (Group 1) were detected in 77.8.0% of HSIL/ASCH and 55.0% of NILM with abnormal colposcopy findings. Six possible carcinogens—HRs (group 2B) were often found in women with normal cytology (14.8%) and mild abnormalities (ASCUS and LSIL), but with lower frequence in HSIL/ASCH lesions (7.1%). HPVs 6 and 11(Group 3) were not found in the cases of HSIL/ASCH. Unclassified HPV types were equally distributed in all cytology groups: 20.7%, 19.1%, 16.3% and 13% of NILM, ASCUS, LSIL and HSIL/ASCH, respectively. Our findings highlight that majority of abnormal Pap test results are caused by Group 1 HPVs among women from our region. Low frequency HPVs of group 2A/2B, especially HSIL/ASCH, supports the conclusion that individual genotypes require consideration of each type as an individual agent. We expect a positive impact of HPV vaccine in reducing HPV-associated cervical lesions among women from Vojvodina province, after establishing vaccination programs in our country.

## Introduction

More than 200 human papillomaviruses (HPVs) genotypes have been molecularly characterized to this day, while only a small fraction of them has the potential to induce malignant transformation of human cells. International Agency for Research on Cancer (IARC) has classified twelve HPV types (16, 18, 31, 33, 35, 39, 45, 51, 52, 56, 58 and 59) from phylogenetic five alpha papillomavirus species α5, α6, α7 and α9 in Group 1 “carcinogenic to humans“, because there is sufficient evidence of carcinogenicity in human population [1,2]. It was estimated that Group 1 genotypes are etiologically associated with more than 98% of all cervical cancers worldwide, 70 to 90% of anal and vaginal cancers, 40% of vulvar cancers, 47% of penile cancers, and 25 to 30% of oropharyngeal cancers. One virus was recognized (Group 2A, HPV68) as “probably carcinogenic to humans”. Recent data indicate that additional HPV types (HPV 26, 53, 66, 67, 70, 73, 82 30, 34, 69, 85, 97) from the alpha papillomavirus species have been identified to achieve single infections in low percentage of cervical cancer tissues. These HPV types are classified as Group 2B “possibly carcinogenic to humans”, based on evolutionary similarity to the known cancer-causing types. Two viruses (HPV 6 and 11) were classified as Group 3 “unclassifiable as to carcinogenicity in humans”. Several other HPVs which may infect the mucosa are signed as unclassified, regarding to their epidemiologic oncogenic risk [3,4].

The human papillomavirus infection is considered to be the main risk factor for cervical cancer development, due to well documented involvement of HPV in malignant transformation of cells [2–4]. Globally observed, the cervical cancer is the fourth most common cancer in women population with an estimated 570,000 new cases in 2018, causing 7.5% of all female cancer deaths [5]. In Serbia, current estimates indicate that every year 1,327 women are diagnosed with cervical cancer and 551 die from this disease. Cervical cancer ranks as the 4^th^ most frequent cancer among women in Serbia and the 2^nd^ most frequent cancer among women between 15 and 44 years of age [5].

Numerous studies have reported that HPV 16 is the most prevalent HPV type in HPV-related cancers worldwide [2–4]. Also, the prevalence of other HPV types shows a wide geographical variability. The knowledge about distribution of HPV genotypes is important for the development of novel diagnostic methods for HPV detection, as well as for the assessment of vaccine efficacy in different geographical regions of the world [6]. Many countries carried out screening and vaccination projects that have been highly effective against cervical cancer. The Government of Serbia has emphasized in several occasions the obligation to drastically reduce morbidity and mortality of HPV related diseases, including cervical cancer. The National Program for Cervical Cancer Screening in the Republic of Serbia has been established in 2012 by targeting women between 25–64 years of age [7]. However, an organized, systematic HPV immunization program, covering certain cohorts (for instance, 12-year-old girls and boys) with governmental funding has not yet been established [8]. In our country, three HPV vaccines have been licensed: bivalent vaccine (2vHPV; Cervarix, GlaxoSmithKline, Serbia), quadrivalent vaccine (4vHPV; Gardasil, Merck Sharp & Dohme D.O.O., Serbia) and nonavalent vaccine (9vHPV; Gardasil 9, Merck Sharp & Dohme D.O.O., Serbia).

In large epidemiological studies, the prevalence of HPVs genotypes from Group 1 has been of the prime interest, while the distribution of probably (2A) and possibly carcinogenic (2B) types among women’s populations in the world is less known [2–4]. The Working Group of IARC has noted that the definition of carcinogenicity is a prerequisite, but it is not sufficient for the inclusion of these HPV types into primary and secondary prevention program [1]. Albeit the fact that the prevalence of different HPVs may vary among countries, current prophylactic vaccines could provide protection from 70 to 90% of cervical cancers.

Only a few studies examined HPV prevalence among women in Serbia, usually women from Belgrade with surrounding areas and from the province of Vojvodina [9–13]. These previous studies were based on the evaluation of HPV prevalence in women with different cervical cytology results, while only one estimated HPV prevalence among women with cervical cancer. All these assays are conducted to detect well known 12–14 high risk HPVs (HR HPV).

The primary objective of this study was to determine the prevalence of different HPV genotypes, including Group 1, 2A, 2B and unclassified types in cervical samples taken from unvaccinated women from Vojvodina province, Serbia. The secondary objectives of this study were to describe and evaluate demographic and clinical parameters according to HPV genotypes and to establish a scientific rationale for implementation of HPV vaccination in Serbia.

## Materials and methods

### Subjects and ethics

This study was conducted within the ongoing project: “Significance of HPV DNA analysis in the prevention of cervical cancer” and the results shown in this paper present the outcome of the analysis conducted from 2016 to 2018. This was a cross-sectional study with convenience type of sampling. Cervical cancer screening was performed by the clinician at the time of pelvic examination, after visualization of cervix. Conventional Pap smears were obtained with the use of cervical Ayer spatulas. Slides were evaluated at the Cytology Laboratory of Department of Gynecology, Community Health Centre Novi Sad, Vojvodina, Serbia and classified according to the Bethesda Classification Diagnosis [14]. The Department of Gynecology, received weekly reports on cytological results from the cytology laboratory. After that, the primary health care personnel called the patients to discuss further treatment and follow-up. HPV tests were suggested to women from 18 to 65 years of age with an abnormal Pap test and to these with normal cytology results with suspicious colposcopic findings (colposcopy is use during the routine gynecological examination in our country). Also, target population was women with normal cervical cytology but in high risk for HPV infection. Participants who met the criteria have got verbal information about the significance of HPV testing by medical staff but only participants who gave an informed consent were included in the study. All women enrolled in the study filled in a questionnaire to facilitate collecting socio-demographic data and information regarding their sexual habits and other risk factors related to acquiring HPV infection.

Samples for HPV DNA analysis were collected using ThinPrep Pap (Hologic Inc.) or eNAT (Copan, Italy) specimen transport medium, according to the manufacturer’s original instructions. The study group consisted of 1,495 women, where 567 women had normal cytology (Negative for Intraepithelial Lesion or Malignancy, NILM), 751 women ASCUS (Atypical Squamous Cell of Undetermined Significance), 136 women LSIL (Low grade Squamous Intraepithelial Lesions) and 41 women had either HSIL (High grade Squamous Intraepithelial Lesions) or ASCH (Atypical Squamous Cells, cannot exclude HSIL). Women enrolled in the study were informed about the research objectives and they all signed an informed consent form. The study protocol was reviewed and approved by the Medical Ethics Committee of the Institute of Public Health of Vojvodina, Novi Sad, Serbia (approval number: 07-1086/3 by 8th September, 2015).

### DNA Extraction and EUROArray HPV test

DNA was extracted from 200 μl specimen by automatic nucleic acid extraction system (SaMag Viral Nucleic Acid Extraction Kit, Sacace, Italy). In each EUROArray HPV test 5 μl of the extracted DNA was used. If amplification was not performed on the same day as extraction, the processed samples were stored at 2–8°C for a maximum of five days or frozen at -20/- 80° C for longer periods. The HPV genotyping assay was performed using the EUROArray HPV test (EUROIMMUN, Luebeck, Germany) according to the manufacturer’s specifications. This test uses an extensive panel of specific primers and probes, to detect thirty genitally relevant HPV types in one reaction. Among them, 18 HPV types (16, 18, 26, 31, 33, 35, 39, 45, 51, 52, 53, 56, 58, 59, 66, 68, 73, 82) included in IARC Group 1, 2A and 2B were regarded to be HR HPV and 12 HPV types (6, 11, 40, 42, 43, 44, 54, 61, 70, 72, 81, 89) included in IARC Group 3 and unclassified types were regarded to be low risk HPVs (LR HPV). Amplification and hybridization steps allowed identification of both the target viral genetic material (E6 /E7 gen) and a fragment of the human Hsp90 gene used as endogenous controls for valid sample extraction and amplification.

Beside positive and negative controls, cross-contamination control (CC-I and CC-II) was incorporated into the test ensure high result security. Chip uses two orientation points (OS I and OS II) for automatic detection of position by EUROArray Scan. The evaluation, interpretation and archiving of results has been fully automated by EUROArrayScan software.

### Statistical analysis

All statistical analyses were performed with software package STATISTICA for Windows 13.0 [15]. The prevalence of HPV infection, genotype distribution, single and multiple HPV infections were analyzed separately and with the chi-square test which was used to compare prevalence of these parameters according to target groups. To examine the relationship between HPV status and behavioral risk factors univariate analyze were performed by calculating odds ratios (OR) and 95% CI. Also, the significance of association between HPV status and cytology as well as between HPV status and age of examined participants was tested by Independence test. In all analyses, probability values of p<0.05 were regarded as significant.

## Results

### Socio-demographic data

A total of 1495 non-vaccinated women (age average: 35 years, age range: 18–65 years) were enrolled in the study. A summary of variables that describe sociodemographic characteristics, lifestyle and sexual habits of participants is provided in Table 1. Statistically significant univariate odds ratio was found for HPV status in group of age 25–34, and in group that had two lifetime sexual partners.

**Table 1:** The profiles of the sociodemographic characteristics of the study group.

Variables	HPV negative	HPV positive	Univariate Odds Ratio (CI 95%)	p-value
	N %	N %		
**Age (years)**				
<25	172 (44.5)	215 (55.5)	1.420 (0.744–2.606)	0.128
25–34	183 (40.6)	268(59.4)	1.664 (0.911–3.041)	0.048*
35–44	201(54.5)	168(45.5)	0.946 (0.517–1.745)	0.434
45–55	154 (63.9)	87(36.1)	0.641 (0.342–1.206)	0.084
>50	25 (53.2)	22 (46.8)	Reference	
**Education level**				
Primary	5 (38.4)	8 (61.6)	1.550 (0.503–4.806)	0.221
Intermediate	416 (49.2)	429 (50.8)	1.602 (0.816–1.231)	0.490
University	314 (48.8)	323 (51.2)	Reference	
**Marital status**				
Single	389 (49.2)	40 3(50.8)	Reference	
Married	270 (51.8)	29 0(48.2)	1.039 (0.837–1.291)	0.363
Divorced/separated	64 (52.1)	594 (7.9)	0.889 (0.608–1.301)	0.237
Widow	12 (60.0)	8 (40.0)	0.643 (0.260–1.591)	0.169
**Age of sexual debut**				
<18	313 (44.4)	391 (55.6)	1.124 (0.922–1.371)	0.124
≥18	422 (47.3)	469 (52.7)	Reference	
**Lifetime sexual partners**				
One	603 (51.5)	567 (48.5)	Reference	
Two	84 (36.4)	147 (63.6)	1.861 (1.390–2.491)	0.000**
≥Three	48 (50.6)	464 (9.4)	1.109 (0.729–1.690)	0.313
**Tobacco users**				
No	520 (49.7)	52 7(50.3)	Reference	
Yes	215 (48.0)	23 3(52.0)	1.069 (0.857–1.334)	0.276

*statistically significant value.

### Prevalence and type-specific distribution of HPV

**Fig 1:**
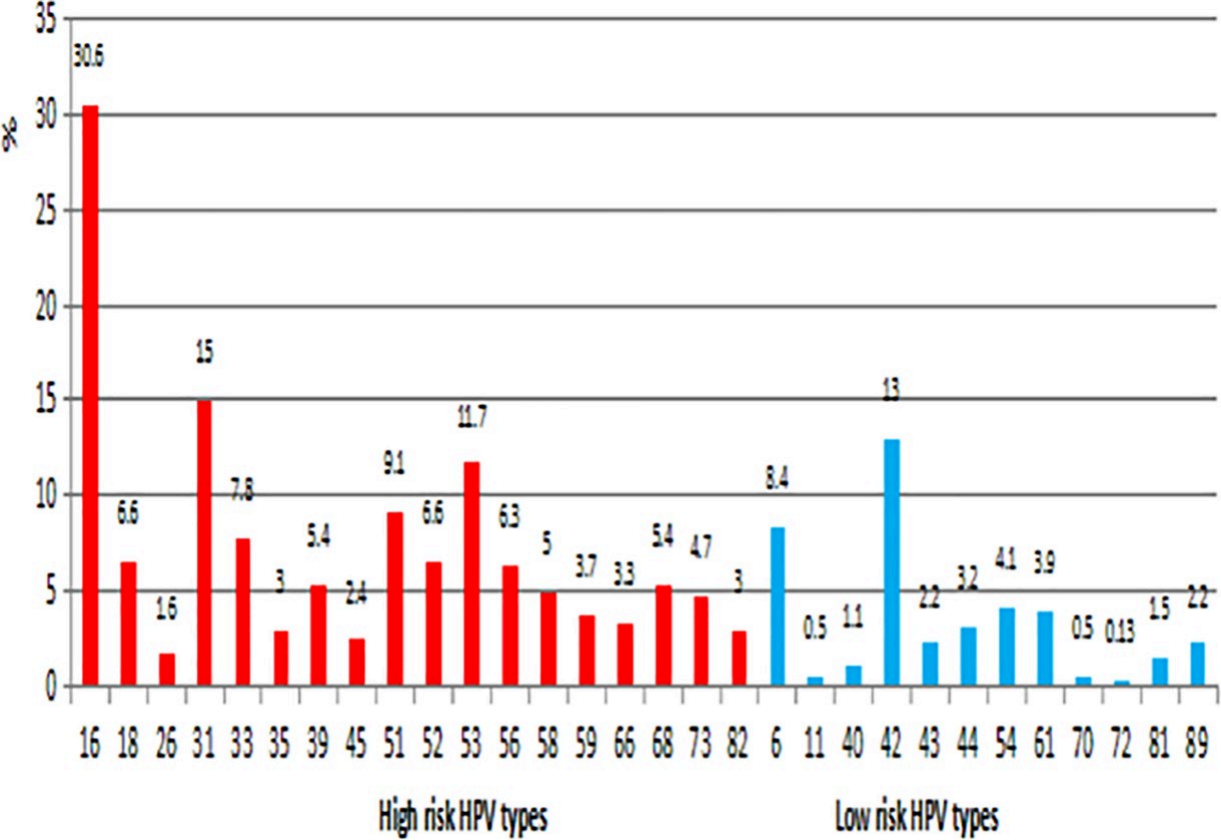
Prevalence of 30 genotypes of HPV among women age 18 to 65 with normal and abnormal cervical cytology.

In the first step, samples were analyzed for the presence of all of 30 genital HPVs where 760 had HPV positive result, so the overall prevalence of HPV in the study population was 50.8%. All of genotypes covered by EUROArray HPV test were identified in study population and the five most prevalent HPV genotypes were HPV 16 (30.6%), HPV 31 (15.0%), HPV 42 (13.0%), HPV 53(11.7%) and HPV 51 (9.1%). Fig 1 illustrates the distribution of the different high risk and low risk HPV genotypes regardless of cytological results and age in analyzed women.

### Distribution of HPV genotypes by cervical cytology and age

Regarding cytological results and age groups, analyzed women were monitored for distribution of high risk and two low risk HPV types, while unclassified HPVs were excluded. Table 2 show HPV DNA types prevalence by age and cervical cytology status. Among women with normal cytology, total HPV infection rate was 37.6% (213 out of 567), while in the group of women with cytological abnormalities the rates were 46.3% (348 out of 751) for ASCUS, 70.6% (96 out of 136) for LSIL and 82.9% (34 out of 41) HISL/ASCH. In HPV-positive cases the highest HR HPV prevalence was reported in HSIL/ASCH (100%). followed by LSIL (95.8%), ASCUS (92.7%) and NILM (84.5%). In contrast to these findings, LR HPVs were more often detected in LSIL (2.1%) while these types were not found in the HSIL/ASCH group.

**Table 2:** HPV DNA prevalence by age and cytological status.

Grouping variables	Total	HPV negative	HPV positive	p-value	HR HPV Positive	LR HPV positive	HR+LR HPV positive	p-value
N (100%)	N (%)	N (%)	N (%)	N (%)	
Cytology								
NILM	567	354 (62.4)	213 (37.6)	0.000**	180 (84.5)	4 (1.9)	29(13.6)	0.000**
ASCUS	751	403 (53.7)	348 (46.3)		323 (92.8)	6 (1.7)	19 (5.5)	
LSIL	136	40 (29.4)	96 (70.6)		90 (93.7)	2 (2.1)	4 (4.2)	
HSIL/ASCH	41	7 (17.1)	34 (82.9)		34 (100)	0 (0.0)	0 (0.0)	
Age (yr)								
<25	387	199(51.4)	188 (48.6)	0.000**	159 (84.6)	5 (2.7)	24 (12.8) (12.7)	0.000**
25–34	451	200 (44.3)	251 (55.7)		231(92.0)	2 (0.8)	18 (7.2)	
35–44	369	216 (58.5)	153 (41.5)		143 (93.4)	3 (2.0)	7 (4.6)	
45–55	241	161 (66.8)	80 (33.2)		76 (95.0)	2 (2.5)	2 (2.5)	
> 55	47	28 (59.6)	19 (40.8)		18 (94.7)	0 (0.0)	1(5.3)	

Values are presented as number and percentage (%).

HR HPV includes participants with HPV types: 16, 18, 26, 31, 33, 35, 39, 45, 51, 52, 53, 56, 58, 59, 66, 68, 73, and 82.

LR HPV includes participants with HPV types: 6, 11.

HR+LR HPV includes participants with found both HR HPV and LR HPV types.

HPV prevalence and distribution of high risk and low risk HPVs types among different age groups are presented in Table 2. The HPV prevalence was shown to be significantly associated with age (p>0.05). The highest prevalence of HPV prevalence was observed among women younger than 25 years (48.6%) and 25–34 years (55.7%). The prevalence of LR HPVs ranged from 2.7% in the youngest age group (<25) and without infection in the oldest age group (> 55).

### Frequency of Group 1, 2A, 2B, 3 and unclassified HPV genotypes related to the cytologic diagnosis

In the next step, HPV DNA positive samples were analyzed according to classification of the IARC. Table 3 shows the relative contribution of the 30 types of HPV overall in correlation to cytological data. By comparison, Group 1 HPV genotypes were detected in 77.8% of HSIL/ASCH and 55.0% of NILM, unlike Group 2B which were more often identified in 15.2% of LSIL. Also, HPV types 16 (30.6%), 31 (15%) and HPV 51(9.1%) were more common in each cytological group. The HPV 16 genotype was markedly higher in the HSIL/ASCH group and was detected by 48.6%.

**Table 3:** The relationship between cytological diagnoses and Group 1, 2A, 2B, 3 and unclassified HPV genotypes among HPV positive women.

Species	HPV genotype	All Sample (n = 760)	NILM (n = 242)	ASCUS (n = 381)	LSIL (n = 101)	HSIL/ASCH (n = 36)
		N (%)	N (%)	N (%)	N (%)	N (%)
**Carcinogens -group 1**						
A9	16	233 (30.6)	66 (27.3)	120 (31.5)	29 (28.7)	18 (50.0)
	31	114 (15.0)	40 (16.5)	51 (13.4)	20 (19.8)	3 (8.3)
	33	59 (7.8)	14 (5.8)	32 (8.4)	10 (9.9)	3 (8.3)
	35	23 (3.0)	8 (3.3)	9 (2.4)	4 (4.0)	2 (2.8)
	52	50 (6.6)	14 (5.8)	25 (6.5)	7 (6.9)	4 (11.1)
	58	38 (5.0)	13 (5.4)	16 (4.2)	5 (5.0)	4 (11.1)
A7	18	50 (6.6)	20 (8.3)	21 (5.5)	7 (6.9)	2 (5.5)
	39	41 (5.4)	17 (7.1)	17 (4.4)	6 (6.0)	1 (2.8)
	45	18 (2.4)	2 (0.8)	12 (3.1)	4 (4.0)	0 (0.0)
	59	28 (3.7)	14 (5.8)	9 (2.4)	5 (5.0)	0 (0.0)
A5	51	69 (9.1)	27 (11.3)	28 (7.3)	10 (9.9)	4 (11.1)
A6	56	48 (6.3)	17 (7.1)	24 (6.3)	6 (6.0)	1 (2.8)
Total group1		**771(58.0)**	**252(55.0)**	**364(57.6)**	**113(61.4)**	**42(77.8)**
**Probable carcinogens -group 2A**						
A7	68	41 (3.1)	9 (2.0)	24 (3.8)	7 (3.8)	1 (1.8)
**Possible carcinogens- group 2B**						
A5	82	23 (3.0)	10 (4.1)	8 (2.1)	4 (4.0)	1 (2.8)
A6	26	9 (1.2)	6 (2.5)	2 (0.52)	1 (1.0)	0 (0.0)
	53	89 (11.7)	28 (11.6)	46 (12.1)	14 (13.8)	1 (2.7)
	66	35 (4.6)	10 (4.1)	19 (4.9)	6 (6.0)	0 (0.0)
A7	70	3 (0.5)	1 (0.4)	1 (0.52)	1 (1.0)	0 (0.0)
A11	73	36 (4.7)	13 (5.4)	19 (4.9)	2 (2.0)	2 (5.5)
Total group 2B		**195(14.6)**	**68(14.8)**	**95(15.0)**	**28(15.2)**	**4(7.4)**
**Not classifiable as to its carcinogenicity (group 3)**						
A10	6	64 (8.4)	33 (12.7)	25 (6.6)	6 (6.0)	0 (0.0)
	11	4 (0.5)	1 (0.4)	3 (0.8)	0 (0.0)	0 (0.0)
Total group 3		**68 (5.2)**	**34 (7.4)**	**28 (4.4)**	**6 (3.3)**	**0 (0.0)**
**Unclassified genotypes**						
A1	42	99 (13.0)	38 (15.8)	43 (11.3)	12 (11.9)	6 (16.7)
A3	61	36 (4.7)	13 (5.4)	16 (4.2)	7 (6.9)	0 (0.0)
	72	1 (0.13)	0 (0.0)	1 (0.0)	0 (0.0)	0 (0.0)
	81	12 (1.5)	5 (2.1)	5 (1.3)	2 (2.0)	0 (0.0)
	89	17 (2.2)	10 (4.2)	6 (1.6)	1 (1.0)	0 (0.0)
A8	40	8 (1.1)	1 (0.41)	5 (1.3)	2 (1.9)	0 (0.0)
	43	16 (2.1)	9 (3.8)	7 (1.8)	0 (0.0)	0 (0.0)
A10	44	24 (3.2)	7 (1.8)	14 (3.7)	2 (2.0)	0 (0.0)
A13	54	41 (5.4)	12 (5.0)	24 (6.3)	4 (4.0)	1 (2.8)
Total Unclassified genotypes		**253 (19.1)**	**95 (20.7)**	**121 (19.1)**	**30 (16.3)**	**7 (13.0)**
**Total in all sample**		**1328**	**458**	**632**	**184**	**54**

Frequencies of cytological groups are presented as numbers and percentages (%). Percentage is calculated in relation to total number of HPV positive participants for specific cytological group with participants with multiple infections included

HPVs 6 and 11 which are not classifiable as carcinogenic (Group 3) were found in 7.4% of NILM, but in no cases in HSIL/ASCH. Six possible carcinogens—HRs (group 2B) were often found in women with normal cytology (14.8%) and mild abnormalities (ASCUS and LSIL), but in lower frequency in HSIL/ASCH lesions (7.4%). Among 2B, HPV 53 showed the highest frequency of 11.7%, regardless of cytological results. Unclassified HPV types were found in similar percentage in all cytology groups: 20.7%, 19.1%, 16.3% and 13.0% of NILM, ASCUS, LSIL and HSIL/ASCH, respectively (Table 3), where HPV 42 (13.0%) was consistently present.

### Prevalence of single and multiple HPV infection

Co-infection with two or more HPV types was observed in 45.6% (347 out of 760) taking into account all HPV DNA positive samples. Women demonstrating positivity for a single HPV genotype accounted for 54.3% (413 out of 760), whereas 25.9% (197 out of 760) of women were positive for dual infections, 11.6% (88 out of 760) for triple infections and 8.2% (62 out of 760) had four or more infections. The percentage of single infection increased significantly from 49.6% in the normal cytological subgroup to 53.5% in the LSIL group and to 61.1% in the HSIL/ASCH group (p<0.05). Dual and triple HPV infections were equally distributed in all of cytology groups, while co-infections with four or more HPV types were not found in the HSIL/ASCH group (Table 4).

**Table 4:** Frequences of single and multiple infections by cytological groups.

HPV positive/cervical cytology	Single HPV Infection n (%)	Multiple HPV infection		
		dual n (%)	triple n (%)	four or more n (%)
NILM (n = 242)	120 (49.6)	63 (26.8)	37 (15.3)	22 (9.1)
ASCUS (n = 381)	217 (56.9)	103 (27.0)	33 (8.7)	28 (7.3)
LSIL (n = 101)	54 (53.5)	23 (22.8)	12 (11.9)	12 (11.9)
HSIL/ASCH (n = 36)	22 (61.1)	8 (22.2)	6 (16.7)	0 (0.0)
**Total (n = 760)**	**413 (54.3)**	**197 (25.9)**	**88 (11.6)**	**62 (8.2)**

**Fig 2:**
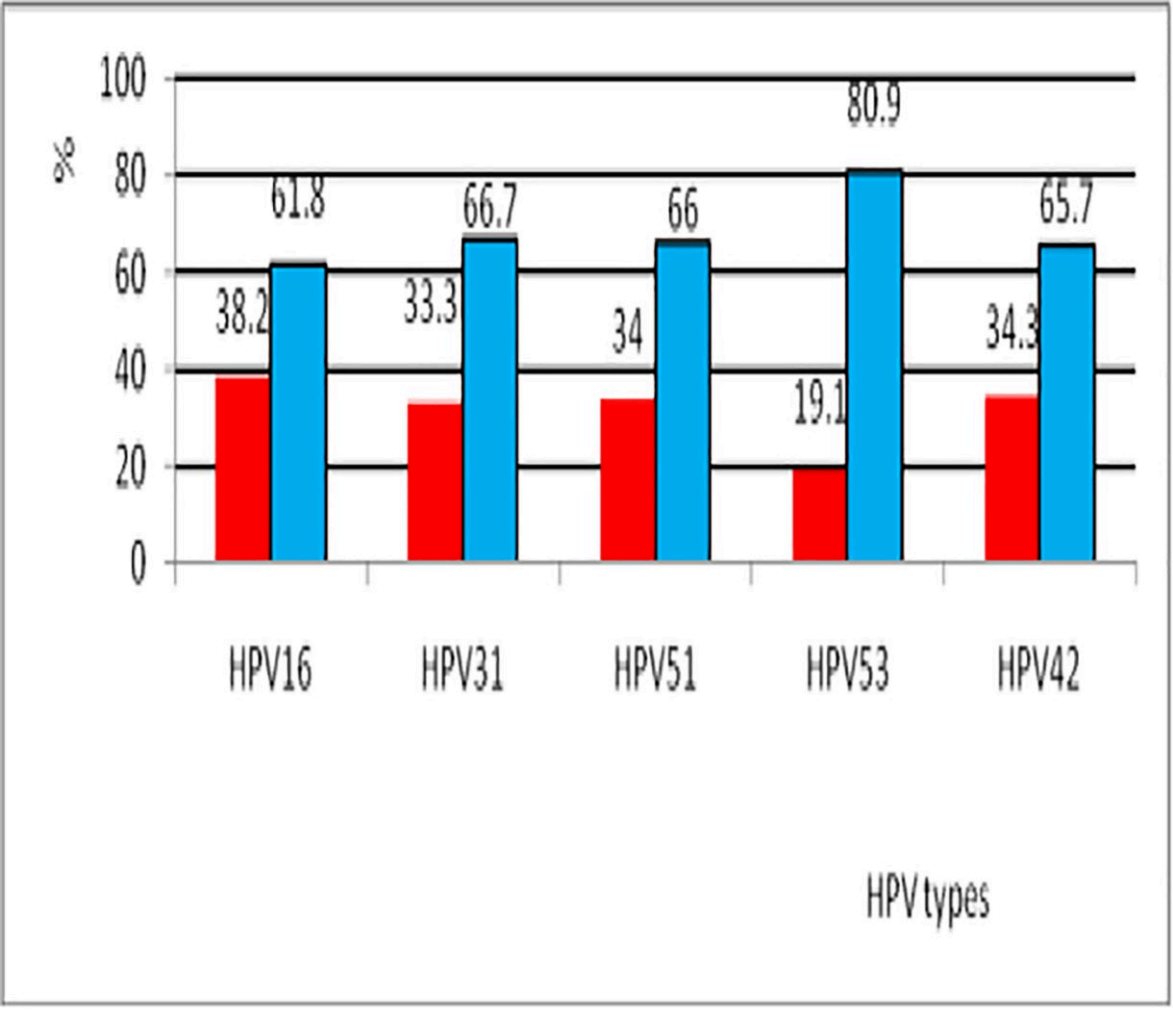
HPV genotypes in cases of single infection and multiple co-infection.

Five most prevalent HPV genotypes (HPV 16, 31, 53, 42 and 51) were analyzed regarding their presence in multiple infections. Among them, HPV 53 was the significantly more common genotype detected in multiple infections (80.9%), followed by HPV 31 (66.7%), HPV 51 (66%), HPV 42 (65.7%) and HPV 16 (61.8%) (Fig 2).

## Discussion

This study provides useful information about the rate of HPV infection and HPV types’ distribution, according to the cytology and age in a cohort of participants from the Province of Vojvodina, Serbia. To the best of our knowledge, this is the first work that examines such a wide spectrum of HPV types in this population. According to EUROArray HPV test of the entire group of 1495 unvaccinated women, the prevalence of HPV DNA was 50.8%. The observed prevalence is not surprising because women with different cytological findings were included in study. These results are similar with our previous studies [11,13] and are consistent with others studies conducted in European countries that reported HPV prevalence ranging from 17.2% to 88.9% among women with different cytological lesions [16,17]. The higher percentage of HPV DNA positive results in women with normal cytological results 37.6% compared to the world average of 11–12% might be attributed to the study cohort which was observed by convenience type of sampling. Patients with NILM cytology were selected for the study because they had abnormal findings on colposcopy. Some of sociodemographic characteristics and sexual behavior of examined population, such as number of lifetime sexual partners and high proportion younger sexually active participants, may be influenced by higher HPV prevalence. In the present study, the association of HPV prevalence with age of participants has been revealed. The highest prevalence of HPV was detected in women younger than 35 years. Such data are expected and correspond with the data reported in other European countries [18,19]. An important reason for relatively high prevalence of HPV probably lies in using HPV DNA test which covers broader range of genotypes. The EUROArray HPV test covers 18 HR HPV types, utilizing a classification system based on novel molecular evidence for oncogenic characteristics of some additional genotypes. Also, twelve genotypes, designated as low-risk HPV types, which share the same transmission pathway with high-risk types can be detected by the EUROArray HPV test. In this study, it was confirmed that all of 30 genotypes covered by EUROArray HPV test circulate among women in Vojvodina province. To evaluate the course and the risk of HPV infection, it is helpful to determine the HPV prevalence at the level of type, which allows discrimination of high- and low risk HPV prevalence and, thus, improve the individual risk assessment of cervical carcinoma. This approach is especially suitable for diagnostic analyses in the framework of HPV screening [6]. In the evaluation of the clinical performance of EUROArray HPV test through the certification monitors VALGENT, this test meets international criteria for its use in cervical cancer screening [20].

Clarification of the contributions of, particularly novel, HPV genotypes causing cervical carcinogenesis will be important to provide an estimate of the potential impact of prophylactic vaccines and cancer screening programs. For this reason, among women in our region, we have analyzed the frequency of each HPV type which IARC marked as carcinogenic, probably carcinogenic and possibly carcinogenic to humans. The specific genotype prevalence with only 12 cancerogenic (Group 1) genotypes, where observed 55.0%, 57.6%, 61.4% and 77.8% in NILM, ASCUS, LSIL and HSIL/ASCH, respectively. The most common 12 HPV types worldwide were consistent with the 12 HPV types classified as Group 1. They were also very frequently present in women of Vojvodina. Among them, HPV 16 was the most prevalent one and HPV 31, 33 51, and 58 were consistently identified, but in lower frequencies. These results correspond with our previous published data [9–13]. However, when observing the prevalence of HPV types from Group 1 among women with the most severe cervical lesions, such as the HSIL and ASCH, we noticed that the most common types were HPV16 (50%), HPV 52 (11.1%), HPV 58 (11.1%), HPV51(11.1%), while HPV 18 were found in 5.5% cases. In carcinoma tissue according to the results of Stamenkovic et al. were found HPV 16 (80.39%), HPV 33 (7.84%), HPV 58 (5.88%), HPV 18 (1.96%), HPV 45 (1.96%) and HPV 53 (1.96%) [10]. HPV 16 and HPV 18 are generally recognized as the most important oncogenic viruses, which account for >70% of all cervical cancers diagnosed worldwide [21–23]. This paper confirms high frequency and oncogenic potential of HPV type 16, as well as lower frequency of HPV 18 in severe cervical lesions and cervical carcinoma [9–13]. The difference between HPV 16 and HPV 18 may be due to the fact that adenocarcinomas are much less common than squamous cell carcinomas. Also, this discrepancy in the HPV type-specific prevalence may be due to the small cohort of HSIL/ACH in this study. Also, it possible that many of the types most frequently found in present study do not necessarily progress to cervical cancer, while HPV 18, which has lower prevalence in Serbia, is more likely to progress to invasive cancer.

Using the EUROArray HPV test, for the first time in the territory of Vojvodina, it has been possible to estimate the prevalence of six types (HPV 26, 53, 66, 70, 73, 82) from group 2A/2B. All of six HPVs were detected within the group of women with normal cytology and mild abnormalities (ASCUS and LSIL). However, in women with high grade lesions as HSIL/ASCH we found only three types: HPV 53 (2.7%), 73 (5.5%) and 82 (2.7%). These results are in concordance with studies conducted by other authors who found that HPVs from group 2A/2B circulate among population women worldwide in different percentage [17,24–26]. However, pHRs had been identified as single HPV infections in about 3% of cervical cancer tissues [21], which raised the question of their potential re-classification [2,27]. Halec et al. reported that eight pHR HPV types, when present as a single infection in cervical cancer, use the same molecular mechanism as any of well-established oncogenic types [21]. However, the authors pointed out that careful estimation of effectiveness and cost-benefit it must be consider before inclusion of these HPV types into population-wide primary and secondary prevention programmes [21]. Based on data from this study, it observed that pHR HPV types circulate among infected women from our region and were founded in low percentage especially among women with HSIL/ASCH lesion. This suggests that pHR HPV types require consideration each type as an individual agent and monitoring their prevalence in future studies.

Interestingly, we have identified nine unclassified genotypes (40, 42, 43, 44, 54 61, 72, 81, 89) with total prevalence of 20.7%, 19,1%, 16.3% and 13.0% in NILM, ASCUS, LSIL and HSIL/ASCH, respectively. It should be noted that HPV 42 is among the top five HPV types detected in present study. This type was consistently frequent in all cytologic subgroups, often in combination with others HPV types. Several studies have reported the presence of this type in various cervical lesions. However, exact role of HPV42 is not well understood [17,25,28]. Data from the study of Guimera et al. suggest that there may be a shift in resolving the role of HPV type 42. According to their results HPV 42 and 70 showing diffuse p16 staining what is characteristic of high-risk HPV types. Authors hypothesized that these types may to be associated with squamous type of malignancy [29].

In this study, multiple infections were found in 45.6%. Such a high percentage of multiple infections raise the issue of cross-contamination. However, EuroArray HPV test use integrated controls such as DNA positive control and cross-contamination control, which ensure the reliability of test results. In addition, EUROArray HPV compared with Anyplex II HPV28 showed high concordance ratio for genotype-specific detection [30]. Observed data are most likely a consequence of various combinations of “unclassified genotypes”, and "possibly carcinogenic to humans HPV types" with HR HPV types. This could be a random pattern, rather than a specific tendency for interaction between these types. Moreover, the random or synergistic interaction of multiple infections are still unresolved [31]. Multiple infections were more frequent in NILM, ASCUS and LSIL including two or more HPV types than in HSIL. Considering the fact that patients infected with multiple HPV types require continuous monitoring to prevent progression of cervical lesions to cancer, screening of genotype and quantity of HPV might provide critical information in the diagnosis of infections and future patient management and prognosis.

One of the objectives of this study was to establish a scientific basis for implementation of HPV vaccination in Serbia. Our results emphasize importance of implementation of HPV vaccination in our region, which would offer direct protection against HPV types that are most commonly associated with cancerogenic alterations and found to circulate most frequently within the population in Vojvodina. According to the present data, the potential impact of the quadrivalent HPV vaccines would be about 50% while the usage of the nonavalent vaccine could prevent more than 84% of cervical precancerous lesions identified in this study.

## Conclusions

Although this study does not reflect all women from province Vojvodina Serbia, the results of this research provide evidence of the high percentage of oncogenic HPV genotypes within infected women group. This data adds support to introduction of HPV vaccine into the national immunization program and integration of HPV DNA tests into in cervical cancer screening programs. Also, data of certain HPV types could not be obtained from previously used methods, since EUROArray HPV test allowed the record of the frequency of “possibly carcinogenic types“, such as 53, 73 and 82, for the first time in our region. It would be of significance to further investigate rare and uncommon HPV genotypes and their possible association with cervical lesions. The data obtained in this study could be of great use in evaluation of the behavior of these infections and associated lesions, as well as for planning of relevant tools for HPV screening and future vaccine implementation.

## References

[1] IARC Working Group on the Evaluation of Carcinogenic Risks to Human. Biological agents. Volume 100 B. A review of human carcinogens. IARC Monogr Eval Carcinog Risks Hum. 2012;100:1–441. . 23189750 PMC4781184

[2] Schiffman M, Clifford G, Buonaguro FM. Classification of weakly carcinogenic human papillomavirus types: addressing the limits of epidemiology at the borderline. Infect Agent Cancer. 2009;4:8. 10.1186/1750-9378-4-8 . 19486508 PMC2694995

[3] Bouvard V, Baan R, Straif K, Grosse Y, Secretan B, El Ghissassi F, et al. A review of human carcinogens-Part B: biological agents. Lancet Oncol. 2009;10:321–322. 10.1016/s1470-2045(09)70096-8 . 19350698

[4] Martel C, Plummer M, Vignat J, Franceschi S. Worldwide burden of cancer attributable to HPV by site, country and HPV type. Int J Cancer. 2017;141:664–670. 10.1002/ijc.30716 . 28369882 PMC5520228

[5] Bruni L, Albero G, Serrano B, Mena M, Gómez D, Muñoz J, et al. Human Papillomavirus and Related Diseases in the World. Summary Report 17 6 2019. [Date Accessed]. Available from: https://www.hpvcentre.net/statistics/reports/XWX.pdf.

[6] Burd EM. Human Papillomavirus Laboratory Testing: the Changing Paradigm. Clin Microbiol Rev. 2016;29:291–319. 10.1128/CMR.00013-15 26912568 PMC4786885

[7] National regulation. Uredba o nacionalnom programu ranog otkrivanja karcinoma grlica materice. Sl. glasnik RS, br. 73/2013 i 83/2013. Available from: http://www.skriningsrbija.rs/files/File/Nacionalni_program_ranog_otkrivanja_karcinoma_grlica_materice.pdf. (in Serbian).

[8] Pravilnik o imunizaciji i nacinu zastite lekovima ("Sl. glasnik RS", br. 82/2017). 2017; Available from: https://www.zczajecar.com/images/dokumenta/doc2017/3.11.-PRAVILNIK-O-IMUNIZACIJI-I-NA%C4%8CINU-ZA%C5%A0TITE-LEKOVIMA-Sl.-glasnik-RS-br.-82-2017.pdf. (in Serbian).

[9] Knezevic A, Aleksic G, Soldatovic I, Banko A, Jovanovic T. Cervical human papillomavirus infection in Serbia: risk factors, prevalence and genotype distribution in women with normal cervical cytology. Arch Biol Sci. 2012;64:1277–1283. 10.2298/ABS1204277K

[10] Stamenkovic M, Knezevic A, Knezevic I, Kuzmanovic I, Karalic D, Milenkovic S, et al. High-risk human papilloma virus genotypes in cervical carcinoma of Serbian women: Distribution and association with pathohistological findings. Biologicals. 2016;44:412–416. 10.1016/j.biologicals.2016.05.001 . 27461126

[11] Kovacevic G, Nikolic N, Jovanovic-Galovic A, Hrnjakovic-Cvjetkovic I, Vuleta D, Patic A, et al. Frequency of twelve carcinogenic human papilloma virus types among women from the South Backa region, Vojvodina, Serbia. Turk J Med Sci. 2016;46:97–104. 10.3906/sag-1410-47 . 27511341

[12] Tasic D, Lazarevic I, Knezevic A, Tasic L, Pikula A, Perisic Z. The impact of environmental and behavioural cofactors on the development of cervical disorders in HR-HPV-infected women in Serbia. Epidemiol Infect. 2018;146:1714–1723. 10.1017/S0950268818001668 . 29923470 PMC9507945

[13] Kovacevic G, Milosevic V, Knezevic P, Knezevic A, Knezevic I, Radovanov J, et al. Prevalence of oncogenic Human papillomavirus and genetic diversity in the L1 gene of HPV16 HPV 18 HPV31 and HPV33 found in women from Vojvodina Province Serbia. Biologicals. 2019;58:57–63. 10.1016/j.biologicals.2019.02.001 . 30795963

[14] Nayar R, Wilbur DC. The pap test and Bethesda 2014. Acta cytol. 2015;59:121–132. 10.1159/000381842 . 25997404

[15] TIBCO, 2017: Software Inc. Statistica (data analysis software system), version 13.

[16] Kjaer SK, Breugelmans G, Munk C, Junge J, Watson M, Iftner T. Population-based prevalence, type- and age-specific distribution of HPV in women before introduction of an HPV-vaccination program in Denmark. Int J Cancer. 2008;123:1864–1870. 10.1002/ijc.23712 . 18661520

[17] Argyri E, Papaspyridakos S, Tsimplaki E, Michala L, Myriokefalitaki E, Papassideri I, et al. A cross sectional study of HPV type prevalence according to age and cytology. BMC Infect Dis. 2013;13:53. 10.1186/1471-2334-13-53 . 23363541 PMC3575232

[18] Smith JS, Melendy A, Rana RK, Pimenta JM. Age-specific prevalence of infection with human papillomavirus in females: a global review. J Adolesc Health. 2008;43:S5–25. 10.1016/j.jadohealth.2008.07.009 . 18809145

[19] Meloni A, Pilia R, Campagna M, Usai A, Masia G, Caredda V, et al. Prevalence and molecular epidemiology of human papillomavirus infection in italian women with cervical cytological abnormalities. J Public Health Res. 2014;3:157. 10.4081/jphr.2014.157 . 25170506 PMC4140382

[20] Viti J, Poljak M, Oštrbenk A, Bhatia R, Alcañiz Boada E, Cornall AM, et al. Validation of EUROArray HPV test using the VALGENT framework. J Clin Virol. 2018;108:38–42. 10.1016/j.jcv.2018.09.005 . 30223253

[21] Halec G, Alemany L, Lloveras B, Schmitt M, Alejo M, Bosch FX, et al. Retrospective International Survey and HPV Time Trends Study Group, Retrospective International Survey and HPV Time Trends Study Group. Pathogenic role of the eight probably/possibly carcinogenic HPV types 26, 53, 66, 67, 68, 70, 73 and 82 in cervical cancer. J Pathol. 2014; 234:441–451. 10.1002/path.4405 . 25043390

[22] Clifford GM, Smith JS, Plummer M, Muñoz N, Franceschi S. Human papillomavirus types in invasive cervical cancer worldwide: a meta-analysis. Br J Cancer. 2003;13:88:63–73. 10.1038/sj.bjc.6600688 . 12556961 PMC2376782

[23] de Sanjose S, Quint WG, Alemany L, Geraets DT, Klaustermeier JE, Lloveras B, et al. Retrospective International Survey and HPV Time Trends Study Group. Human papillomavirus genotype attribution in invasive cervical cancer: a retrospective cross-sectional worldwide study. Lancet Oncol. 2010;11:1048–1056. 10.1016/S1470-2045(10)70230-8 . 20952254

[24] Otero-Motta AP, Ordonez JL, Gonzalez-Celador RA, Rivas B, Garcia Macias MD, Bullon A, et al. Prevalence of human papillomavirus genotypes in cytologic abnormalities from unvaccinated women living in north‐western Spain. Apmis. 2011;119:204–15. 10.1111/j.1600-0463.2010.02711.x . 21284738

[25] Martins TR, de Oliveira CM, Rosa LR, de Campos Centrone C, Rodrigues CL, Villa LL, et al. HPV genotype distribution in Brazilian women with and without cervical lesions: correlation to cytological data. Virol J. 2016;13:138. 10.1186/s12985-016-0594-3 . 27515763 PMC4982268

[26] Herraez-Hernandez E, Alvarez-Perez M, Navarro-Bustos G, Esquivias J, Alonso S, Aneiros-Fernandez J, et al. HPV Direct Flow CHIP: A new human papillomavirus genotyping method based on direct PCR from crude-cell extracts. J Virol Methods. 2013;193:9–17. 10.1016/j.jviromet.2013.04.018 . 23680093

[27] Clifford GM, Howell‐Jones R, Franceschi S. Judging the carcinogenicity of human papillomavirus types by single/multiple infection ratio in cervical cancer. Int J Cancer. 2011;129:1792–4. 10.1002/ijc.25833 . 21140454

[28] Annunziata C, Stellato G, Greggi S, Sanna V, Curcio MP, Losito S, et al. Prevalence of "unclassified" HPV genotypes among women with abnormal cytology. Infect Agent Cancer. 2018;13:26. 10.1186/s13027-018-0199-0 . 30061920 PMC6056927

[29] Guimerà N, Lloveras B, Lindeman J, Alemany L, van de Sandt M, Alejo M, et al. The occasional role of low-risk human papillomaviruses 6, 11, 42, 44, and 70 in anogenital carcinoma defined by laser capture microdissection/PCR methodology: results from a global study. Am J Surg Pathol. 2013;37:1299–310. 10.1097/PAS.0b013e31828b6be4 . 24076770

[30] Latsuzbaia A, Tapp J, Nguyen T, Fischer M, Arbyn M, Weyers S, et al. Analytical performance evaluation of Anyplex II HPV28 and Euroarray HPV for genotyping of cervical samples. Diagn Microbiol Infect Dis. 2016;85:318–322. 10.1016/j.diagmicrobio.2016.04.011 . 27156793

[31] Hajia M, Sohrabi. Possible Synergistic Interactions Among Multiple HPV Genotypes in Women Suffering from Genital Neoplasia. Asian Pac J Cancer Prev. 2018;27:785–789. 10.22034/APJCP.2018.19.3.785 . 29582635 PMC5980856

